# Activation of the PI3K/mTOR Pathway Is Involved in Cystic Proliferation of Cholangiocytes of the PCK Rat

**DOI:** 10.1371/journal.pone.0087660

**Published:** 2014-01-30

**Authors:** Xiang Shan Ren, Yasunori Sato, Kenichi Harada, Motoko Sasaki, Shinichi Furubo, Jing Yu Song, Yasuni Nakanuma

**Affiliations:** 1 Department of Human Pathology, Kanazawa University Graduate School of Medicine, Kanazawa, Japan; 2 Department of Pathology, Yanbian University College of Medicine, Yanji-city, China; 3 Department of Pathology, Shizuoka Cancer Center, Shizuoka, Japan; H. Lee Moffitt Cancer Center & Research Institute, United States of America

## Abstract

The polycystic kidney (PCK) rat is an animal model of Caroli’s disease as well as autosomal recessive polycystic kidney disease (ARPKD). The signaling pathways involving the mammalian target of rapamycin (mTOR) are aberrantly activated in ARPKD. This study investigated the effects of inhibitors for the cell signaling pathways including mTOR on cholangiocyte proliferation of the PCK rat. Cultured PCK cholangiocytes were treated with rapamycin and everolimus [inhibitors of mTOR complex 1 (mTOC1)], LY294002 [an inhibitor of phosphatidylinositol 3-kinase (PI3K)] and NVP-BEZ235 (an inhibitor of PI3K and mTORC1/2), and the cell proliferative activity was determined in relation to autophagy and apoptosis. The expression of phosphorylated (p)-mTOR, p-Akt, and PI3K was increased in PCK cholangiocytes compared to normal cholangiocytes. All inhibitors significantly inhibited the cell proliferative activity of PCK cholangiocytes, where NVP-BEZ235 had the most prominent effect. NVP-BEZ235, but not rapamycin and everolimus, further inhibited biliary cyst formation in the three-dimensional cell culture system. Rapamycin and everolimus induced apoptosis in PCK cholangiocytes, whereas NVP-BEZ235 inhibited cholangiocyte apoptosis. Notably, the autophagic response was significantly induced following the treatment with NVP-BEZ235, but not rapamycin and everolimus. Inhibition of autophagy using siRNA against protein-light chain3 and 3-methyladenine significantly increased the cell proliferative activity of PCK cholangiocytes treated with NVP-BEZ235. In vivo, treatment of the PCK rat with NVP-BEZ235 attenuated cystic dilatation of the intrahepatic bile ducts, whereas renal cyst development was unaffected. These results suggest that the aberrant activation of the PI3K/mTOR pathway is involved in cystic proliferation of cholangiocytes of the PCK rat, and inhibition of the pathway can reduce cholangiocyte proliferation via the mechanism involving apoptosis and/or autophagy.

## Introduction

Caroli’s disease is characterized by the progressive, multiple cystic dilatation of intrahepatic bile ducts, and is frequently associated with portal fibrosis corresponding to congenital hepatic fibrosis [1]. It belongs to a group of congenital hepatorenal fibrocystic syndrome, and is a hepatic manifestation of autosomal recessive polycystic kidney disease (ARPKD) [2]. The polycystic kidney (PCK) rat is an established animal model of Caroli’s disease with congenital hepatic fibrosis as well as ARPKD [3]. Using the PCK rat, potential therapeutic strategies for Caroli’s disease and ARPKD have been analyzed. However, no effective therapy applicable to human disease has been established [4].

The mammalian target of rapamycin (mTOR) has attracted attention because of its involvement in a variety of diseases including cancer [5]. The mTOR functions as two distinct multiprotein complexes, mTOR complex 1 (mTORC1) and complex 2 (mTORC1). The mTORC1 phosphorylates S6 kinase (S6) and eukaryotic initiation factor 4E-binding protein-1 (4E-BP1), and regulates cell growth, proliferation, and survival. The mTORC2 has been suggested that it lies downstream of phosphatidylinositol 3-kinase (PI3K) signaling and phosphorylates Akt on Ser473, although it is less well studied in contrast to mTORC1.

Rapamycin and its analog, everolimus, are inhibitors of mTORC1, and have antitumor effects in various cancers by inhibiting cell proliferation, and by affecting apoptosis and autophagy. The rapamycin-sensitive mTORC1 contains phosphorylated (p)-Ser2448, which is consistent with Ser2448 phosphorylation being sensitive to acute rapamycin treatment. The rapamycin-insensitive mTORC2 complex contains p-Ser2481, which is consistent with Ser2481 being a rapamycin-insensitive autophosphorylation site [6].

Rapamycin have been shown to be effective in rodent models of autosomal dominant PKD (ADPKD), which is consistent with the findings showing that the PI3K/Akt/mTOR pathway is aberrantly activated in ADPKD [7]–[11]. However, in clinical trials, rapamycin and everolimus have not shown benefit in patients with ADPKD [12], [13]. Similarly, rapamycin failed to attenuate progression of kidney and liver lesions in the PCK rat in vivo, although the activation of Akt/mTOR pathway has been observed in patients with ARPKD [14], [15]. Thus, the clinical application of mTORC1 inhibitors to PKD patients seems to be limited of use.

Recently, multi-targeting approach against the PI3K/mTOR pathway has provided novel tools for elucidation of the roles of mTOR and mTOR-based therapeutic strategy. NVP-BEZ235 belongs to the class of imidazoquinolines, and is a novel inhibitor of PI3K and mTORC1/2 [5]. In a certain type of human neoplastic cells, NVP-BEZ235 suppresses cell proliferation by inducing apoptosis or autophagy [16], [17], but its therapeutic benefits for the pathogenesis of PKD have not been tested.

Disturbance in the balance between apoptosis and cell proliferation is a pathologic feature of PKD. Increased apoptosis has been observed in human ADPKD as well as the rodent models of PKD [3], [18]–[21]. Activation of caspase signaling and dysregulation of antiapoptotic bcl-2 protein have been described in PKD. Recently, the role of autophagy has been investigated in mouse models of PKD [22]. However, the pathologic significance of autophagy in the PCK rat is largely unknown.

In this study, the effects of the PI3K and/or mTORC1/2 inhibitors were investigated using the PCK rat to clarify the mechanism of cystic dilatation of the intrahepatic bile ducts and their therapeutic usefulness for the inhibition of bile duct dilatation, particularly focusing on the cell proliferative activity in relation to apoptotic and autophagic responses.

## Materials and Methods

### Reagents and Antibodies

Rapamycin and everolimus were purchased from Sigma-Aldrich (St. Louis, MO), and 3-methyladenine (3MA) and LY294002 were from Calbiochem (San Diego, CA). Antibodies for Akt, p-Akt (Ser473), S6, p-S6 (Ser235/236), mTOR, p-mTOR (Ser2448), p-mTOR (Ser2481), p-ERK1/2, PI3K p110α, PI3K p85, 4E-BP1, p-4E-BP1(T37/46), protein-light chain3 (LC3), cleaved caspase 3, and Rictor were purchased from Cell Signaling Technology (Danvers, MA). Antibodies against p-mTOR (Ser2448), LC3, Ki-67 and β-actin were purchased from Santa Cruz Biotechnology Inc. (Santa Cruz, CA), Nanotools (Munich, Germany), Nichirei (Tokyo, Japan) and Abcam (Cambridge, UK), respectively.

### Animals

The PCK rats were maintained at the laboratory animal institute of Kanazawa University Graduate School of Medicine. Normal (Crj:CD) rats were purchased from Charles River Japan (Sagamihara, Japan). The protocol was approved by the committee of Institute for Experimental Animals, Kanazawa University Advanced Science Research Center (Permit Number: AP-122551). Animal studies were performed in accordance with the guidelines for the Care and Use of laboratory Animals at Takara-machi Campus of Kanazawa University.

### Liver Specimens

For immunohistochemical analysis, livers were removed from normal and PCK rats. The liver tissues were immersed in 10% formalin neutral buffer solution (pH 7.4), and embedded in paraffin. More than 10 serial sections, 4 µm thick, were cut from each paraffin block, and were subjected to the analysis.

### Cell Culture of Cholangiocytes

Cholangiocytes were isolated, purified, and cultured from the intrahepatic large bile ducts of normal and PCK and rats as described previously [23], [24]. The cells were set on cell culture dishes covered with a standard medium composed of Dulbecco’s modified Eagle’s medium/F-12 (Gibco, Grand Island, NY) containing 10% bovine growth serum (HyClone, Logan, UT), 5 µmol/L forskolin (Wako Pure Chemical Industries, Osaka, Japan), 20 ng/ml of epidermal growth factor (Upstate Biotechnology, Lake Placid, NY) and 1% antibiotics-antimycotic (Life Technologies) at 37^o^C in an atmosphere of 5% CO_2_.

### Cell Proliferation Assay

Proliferative activity of cholangiocytes was determined using the WST-1 assay according to the manufacturer’s instructions (Roche, Mannheim, Germany). The cells were seeded in 96-well tissue culture plates and maintained for 24 hours with the standard medium. Then the cells were treated with rapamycin, everolimus, NVP-BEZ235 and LY294002 at the concentrations and at the intervals indicated. The WST-1 reagent was added and incubated for 1 hour before reading the plate. Each assay was conducted in six sets.

### Three-dimensional Cell Culture

Biliary cyst formation were examined using the three-dimensional cell culture system with the use of the growth factor reduced Matrigel (BD Biosciences, San Jose, CA). On day 6 after the beginning of the cell culture, the culture medium was changed to that containing rapamycin (100 ng/ml), everolimus (100 nM) and NVP-BEZ235 (100 nM). Incubation was continued for further 72 hours. The morphological changes were recorded using a digital camera under phase-contrast microscopy. More than 6 fields at x40 magnification were recorded for each experimental group, and the size of well-developed cysts was analyzed within the field. The size of the same cyst was determined at day 0 (before the treatment) and day 3 (72 hours after the treatment) on the digital images, and the growth rate was calculated.

### Quantitative Real-time PCR

Reverse transcriptase-PCR was performed using total RNA (1 µg) extracted from the cholangiocytes. Total RNA was extracted using an RNA extraction kit (RNeasy mini; Qiagen, Tokyo, Japan) and was used to synthesize cDNA with reverse transcriptase (ReverTra Ace; Toyobo, Osaka, Japan). Quantitative real-time PCR was performed according to a standard protocol using the SYBR Green PCR Master Mix (Toyobo Co.) and Mx3000P&Mx3005P Real-Time QPCR System (Applied Biosystems, CA). The sequences of the primers (5′–3′) used were: bcl-2; forward, CTGGCATCTTCTCCTTCCAG; reverse, CGGTAGCGACGAGAGAAGTC: glyceraldehyde-3-phosphate dehydrogenase (GAPDH); forward, GAGTCAACGGATTTGGTCGT; reverse, TTGATTTTGGAGGGATCTC. Cycling conditions were incubated at 62°C for 2 minutes, 95°C for 10 minutes, 40 cycles of 95°C for 15 seconds and 60°C for 1 minute. Fold difference compared with GAPDH was calculated. Each assay was conducted in three sets.

### Western Blot Analysis

Total proteins were extracted from the cells using T-PER protein extraction reagent (Pierce Chemical Co., Rockford, IL). The protein was subjected to 10% SDS-polyacrylamide electrophoresis, and then electrophoretically transferred on to a nitrocellulose membrane. The membrane was incubated with primary antibodies against Akt (1∶1000), p-Akt (Ser473) (1∶1000), S6 (1∶500), p-S6 (1∶500), mTOR (1∶500), p-mTOR (Ser2448) (1∶500; Cell Signaling Technology), p-mTOR (Ser2481) (1∶500), p-ERK1/2 (1∶500), PI3K p110α (1∶500), PI3K p85 (1∶500), 4E-BP1 (1∶1000), p-4E-BP1 (T37/46) (1∶1000), cleaved caspase 3 (1∶400), LC3 (1∶500; Cell Signaling Technology), and Rictor (1∶500). The protein expression was detected using an EnVision+system (DakoCytomation, Glostrup, Denmark). 3,3′-diaminobenzidine tetrahydrochloride (DAB) was used as the chromogen. Semiquantitative analysis of the results was performed for three independent experiments using NIH J image software (National Institutes of Health, Bethesda, MD).

### Apoptosis Assay

Flow cytometric analysis of apoptosis was performed by the quantitative detection of phosphatidylserine of the cell surface using Annexin V/FITC and PI apoptosis detection kit (BD Biosciences, San Jose, CA). Cultured cholangiocytes were treated with rapamycin (100 ng/ml), everolimus (100 nM), and NVP-BEZ235 (500 nM) for 24 hours. The cells were washed twice with cold phosphate-buffered saline, and suspended in binding buffer. The cell suspension (100 µl) was incubated with Annexin V-FITC (5 µl) and propidium iodide (PI) (2 µl) for 15 minutes at room temperature. Then binding buffer (400 µl) was added in the cell suspension, and the samples were analyzed within 1 hour using flow cytometer (JASAN, Bay bioscience, Kobe, Japan). Apoptotic cells were expressed by the percentage of Annexin V-FITC positive-, PI negative-cells of total gated cells. The experiments were performed in three sets for each condition. Apoptosis positive control cells were treated with 200 µM H_2_O_2_ for 24 hours.

For the detection of apoptotic cells in paraffin-embedded tissue sections, a terminal dUTP nick-end labeling (TUNEL) method was used. After proteinase K digestion and endogenous peroxidase blocking, the sections were stained by using a commercial kit (TdT-FragEL DNA fragmentation detection kit, Calbiochem). After color development with DAB, sections were counterstained with methyl green.

### Transfection of siRNA

Synthetic siRNA for LC3, caspase 3 and Rictor, and non-silencing siRNA (negative control) were purchased from Qiagen (Tokyo, Japan). Transfections of siRNA were performed using HiPerFect Transfection Reagent (Qiagen) according to the manufacturer’s instructions. Cholangiocytes were incubated for 24 hour with the standard medium. After removal of the medium, the cells were incubated with Dulbecco’s modified Eagle’s medium/F-12 (Gibco) containing premixed siRNA (10 nM) and 3 µl of HiPerFect Transfection Reagent, and were further incubated for 72 hours.

### Immunofluorescence Confocal Microscopy

Cholangiocytes were seeded in slide chamber, and were treated with rapamycin (100 ng/ml), everolimus (100 nM), and NVP-BEZ235 (500 nM) for 24 hours. They were fixed with 4% paraformaldehyde for 15 minutes, and were permeabilized for 10 minutes with 0.1% Triton X-100. After blocking, the cells were incubated with a primary antibody against LC3 (1∶200; Cell Signaling Technology) for overnight at 4°C. Alexa-488 (10 µg/ml; Molecular Probes, Eugene, OR) was used as a secondary antibody, and nuclei were stained with 4′6-diamidino-2-phenylindole. Positive control of autophagy was the cells incubated with serum-free medium for 24 hours.

### Immunohistochemistry

After deparaffinization of the sections, antigen retrieval was performed by microwaving in 10 mmol/L citrate buffer (pH 6.0) for p-Akt, p-S6 and LC3 staining, and by heating in Tris-ethylenediaminetetraacetic acid buffer (pH 9.0) with a pressure cooker for p-mTOR and Ki-67 staining. After blocking the endogenous peroxidase, the sections were incubated overnight at 4°C with anti-p-mTOR (Ser2448) (1∶20; Santa Cruz Biotechnology, Inc.), anti-p-Akt (Ser473) (1∶25), anti-p-S6 (1∶200), anti-LC3 (1∶100; Nanotools) and anti-Ki-67 (pre-diluted) antibodies. Then the sections were incubated with the secondary antibody conjugated to the peroxidase-labeled polymer, EnVison+system (DakoCytomation). Color development was performed using DAB, and the sections were counterstained with hematoxylin. Control sections were evaluated by substitution of the primary antibodies with nonimmunized serum without signal detection.

### In vivo Administration of NVP-BEZ235

A total of 22 male rats were used. At 4 weeks of age, 11 normal and 11 PCK rats were divided into control and experimental groups. The experimental groups were intraperitoneally administrated 20 mg/kg NVP-BEZ235 daily between 4 and 8 weeks of age. The dosage was determined based on the previous report [25]. The control group received vehicle (2% Tween 80 and 1% methylcellulose in water) alone. At 8 weeks of age, rats were weighed and anesthetized with diethyl ether. Blood was obtained by puncture of inferior vena cava for hematological analysis. The liver and kidney were weighed and immersed in 10% formalin neutral buffer solution (pH 7.4), and the tissues were embedded in paraffin for histological analysis.

### Histological Assessment

Paraffin-embedded sections were prepared for the liver and kidney, and whole tissue sections were used to measure cyst volumes and liver fibrosis. Cyst volumes of the liver and kidney were assessed using hematoxylin-eosin stained sections. Liver fibrosis was assessed using picrosirius red staining. Stained sections were visualized under a light microscope, and the digital images were acquired and reproduced on a computer. Image analysis was performed in software using Adobe Photoshop (Adobe Systems Incorporated, San Jose, CA). A color threshold was applied at a level that separated cysts from non-cystic tissue or the picrosirius red stained material from the background to calculate the volume of the cysts or fibrosis. The areas of interest were expressed as a percentage of the total tissue.

Evaluation of apoptosis was performed using liver sections stained using the TUNEL method. More than 500 biliary epithelial cells were surveyed in the sections, and the percentage of biliary epithelial cells positive for TUNEL was expressed as the TUNEL-labeling index.

To evaluate the cell proliferative activity, Ki-67 protein-positive signals were similarly counted for biliary epithelial cells of the Ki-67-immunostained liver sections, and the percentage of biliary epithelial cells positive for Ki-67 protein were expressed as the Ki-67-labeling index.

### Statistics

The mean ± SD was calculated for all parameters. Statistical differences were determined using the T-test and analysis of variance. A *p* value <0.05 was accepted as the level of statistical significance.

## Results

### Overexpression of p-mTOR, p-Akt, p-S6 and PI3K in PCK Cholangiocytes

**Figure 1:**
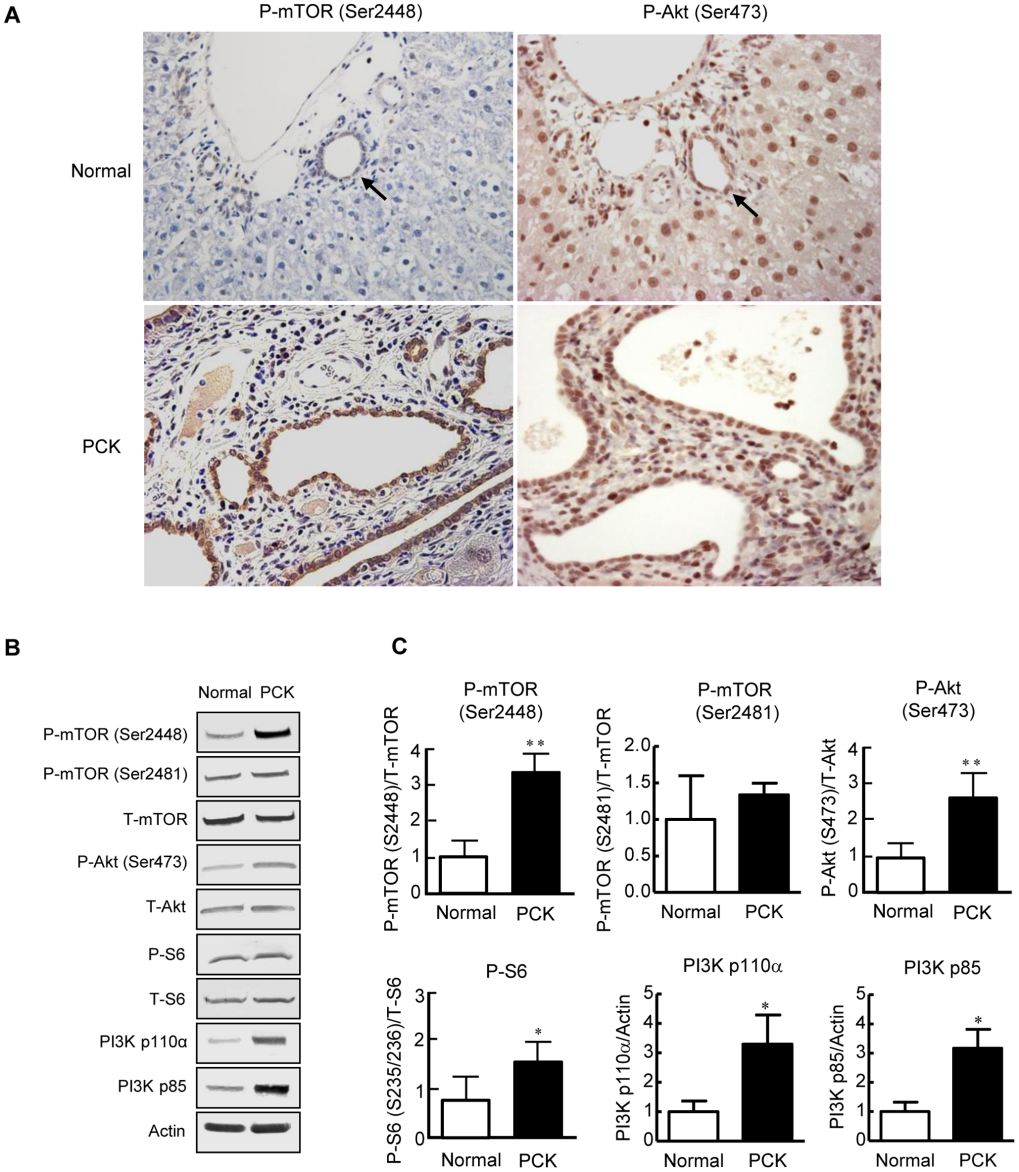
Overexpression of p-mTOR, p-Akt, p-S6, and PI3K in PCK cholangiocytes. The immunohistochemical expression of p-mTOR (Ser2448) and p-Akt (Ser473) was examined using liver sections of 10-month-old rats. Increased expression of p-mTOR (Ser2448) and p-Akt (Ser473) was observed in bile duct epithelium of the PCK liver compared to that of the normal liver (A). Western blot analysis using protein extracts from cultured cholangiocytes showed that the expression p-mTOR (Ser2448), p-mTOR (Ser2481), p-Akt (Ser473), p-S6, PI3K p110α, and PI3K p85 was increased in PCK cholangiocytes compared to normal cholangiocytes (B). The results of the semiquantitative analysis of Western blotting are shown in C. Arrows indicate interlobular bile ducts of the normal liver. *, *p*<0.01; **, *p*<0.05 (vs. normal cholangiocytes). Original magnifications; x400 (A).

The immunohistochemical expression of p-mTOR (Ser2448) and p-Akt (Ser473) was examined using liver sections of 10-month-old rats. In the normal rat, the expression of p-mTOR (Ser2448) in bile duct epithelium was faint or negligible, and that of p-Akt (Ser473) was weak (Figure 1A, arrows). In the PCK liver, the expression of both p-mTOR (Ser2448) and p-Akt (Ser473) in bile duct epithelium was markedly increased (Figure 1A).

Western blot analysis showed that the expression p-mTOR (Ser2448), p-mTOR (Ser2481), p-Akt (Ser473), p-S6, PI3K p100α, and PI3K p85 was increased in cultured PCK cholangiocytes compared to normal cholangiocytes (Figure 1B and 1C), where the expression p-mTOR (Ser2448) was indicative of the activation of mTORC1, and p-mTOR (Ser2481) and p-Akt (Ser473) were indicative of the activation of mTORC2. The expression of p-ERK1/2 was not significantly different between normal and PCK cholangiocytes (data not shown).

### In vitro Effects of PI3K and mTOR Inhibitors on Cholangiocytes

**Figure 2:**
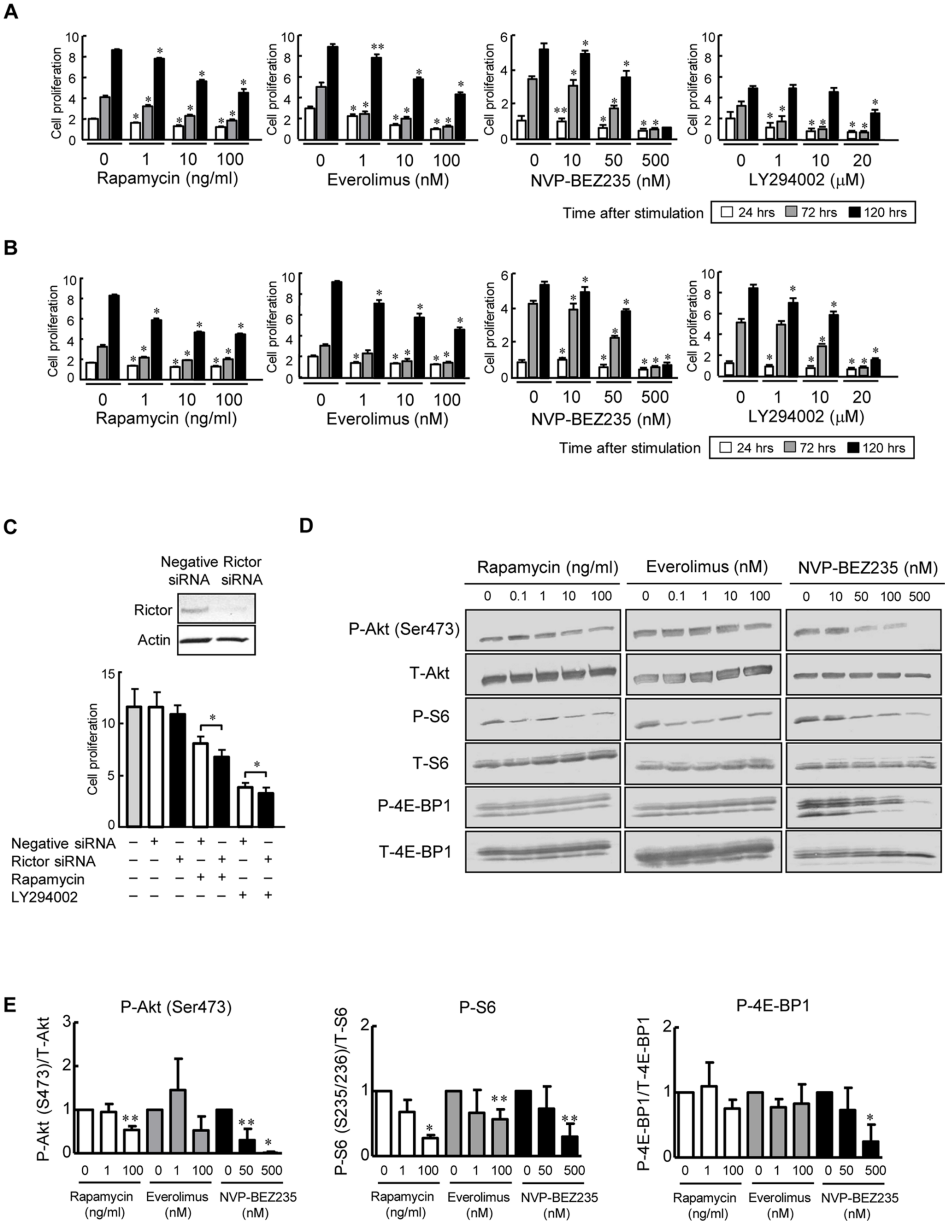
In vitro effects of PI3K and mTOR inhibitors on cholangiocytes. Normal and PCK cholangiocytes were treated with the PI3K and/or mTOR inhibitors, and the cell proliferative activity was determined using the WST-1 assay as described in the Materials and Methods. Rapamycin, everolimus, NVP-BEZ235 and LY294002 significantly inhibited the cell proliferative activity of normal (A) and PCK (B) cholangiocytes in a dose-dependent fashion, where the most prominent inhibitory effect was observed in both cholangiocytes treated with NVP-BEZ235 for 120 hours. Rictor siRNA significantly reduced the cell proliferative activity of the PCK cholangiocytes when it was used in combination with rapamycin and LY 294002 (C). Western blot analysis showed that the expression of p-Akt (Se473) and p-S6 was reduced following 24 hour-treatment with rapamycin, everolimus and NVP-BEZ235 in a dose-dependent fashion in PCK cholangiocytes (D). The results of the semiquantitative analysis of Western blotting are shown in E. Note that the expression of p-Akt (Ser473) and p-4E-BP1 was markedly decreased in PCK cholangiocytes following the treatment with 500 nM NVP-BEZ235 (D and E). *, *p*<0.01; **, *p*<0.05 (vs. untreated control).

Normal and PCK cholangiocytes were treated with rapamycin (mTORC1 inhibitor), everolimus (mTORC1 inhibitor), NVP-BEZ235 (PI3K and mTORC1/2 inhibitor) and LY294002 (PI3K inhibitor), and the cell proliferative activity was determined using the WST-1 assay. Each inhibitor significantly inhibited the cell proliferative activity of normal (Figure 2A) and PCK (Figure 2B) cholangiocytes in a dose-dependent fashion. Above all, NVP-BEZ235 had most prominent inhibitory effects on the cell proliferative activity of both cholangiocytes, and at the concentration of 500 nM, it reduced the cell proliferative activity of both cholangiocytes to less than 30% compared to that of untreated groups at 120 hours (Figure 2A and 2B). Higher concentrations of rapamycin (10 µg/ml) and everolimus (10 µM) showed almost same inhibitory effects on the cell proliferative activity as those seen in 100 ng/ml rapamycin and 100 nM everolimus, respectively (data not shown).

To address the role of mTORC2 in cholangiocyte proliferation, the experiment of depletion of Rictor using siRNA was performed. Western blot analysis confirmed the gene silencing for Rictor was efficiently conducted (Figure 2C, upper panel). Although the Rictor siRNA alone did not affect the cell proliferative activity of PCK cholangiocytes, the cell proliferative activity was further reduced when the siRNA was used in combination with rapamycin and LY294002 (Figure 2C). Thus, mTORC2 was partially involved in the increased cell proliferation of PCK cholangiocytes.

Western blot analysis showed that the expression of p-Akt (Se473) and p-S6 was reduced following the treatment with rapamycin, everolimus and NVP-BEZ235 in a dose-dependent fashion in PCK cholangiocytes, and NVP-BEZ235 further reduced the expression of p-4E-BP1 (Figure 2C and 2D). Higher concentration (500 nM) of NVP-BEZ235 almost completely diminished the expression of p-Akt (Ser473).

### Effects of mTOR Inhibitors on Cholangiocyte Apoptosis in vitro

**Figure 3:**
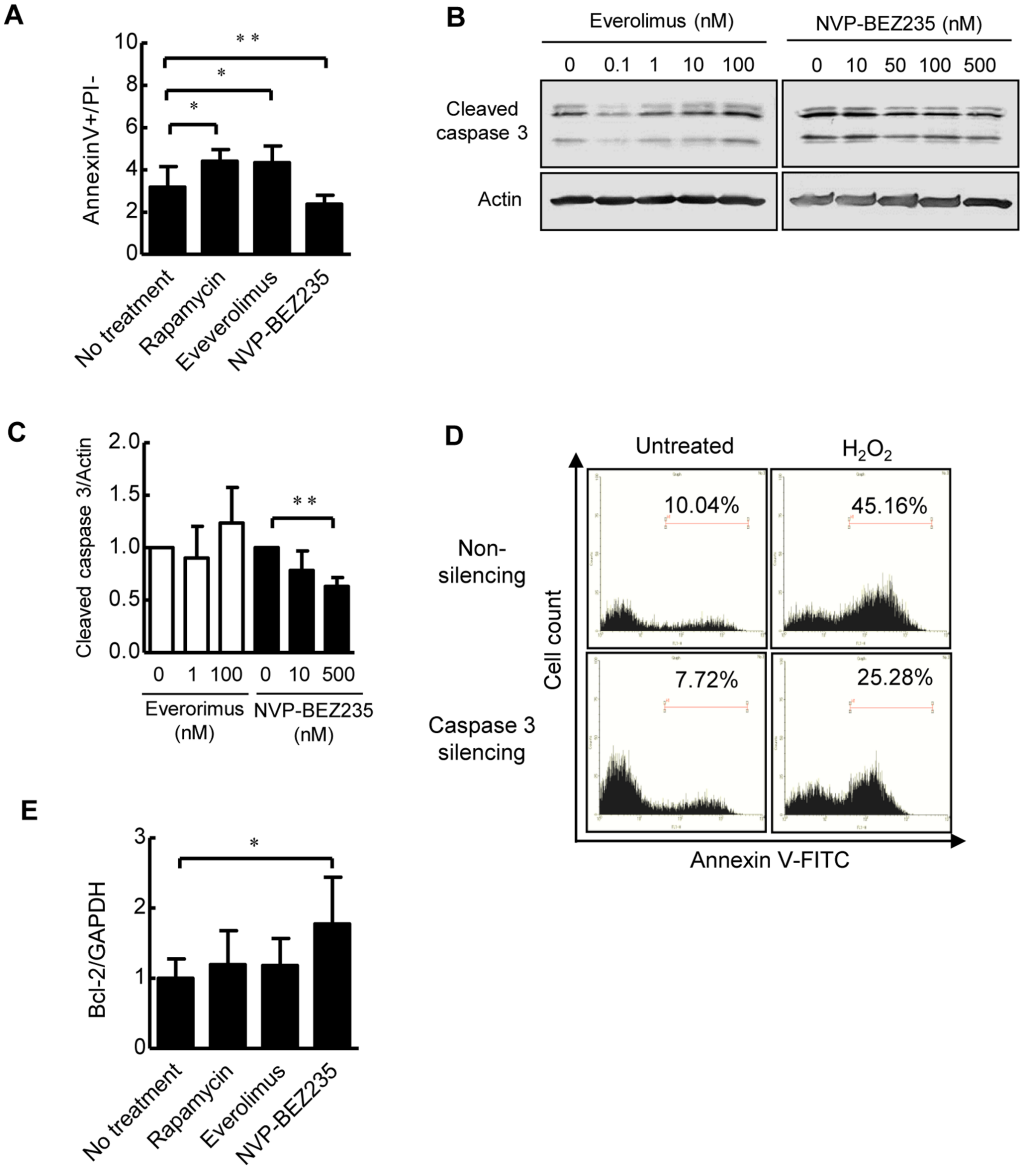
Effects of mTOR inhibitors on cholangiocyte apoptosis in Apoptosis was determined following the treatment of PCK cholangiocytes with rapamycin (100 ng/ml), everolimus (100 nM) and NVP-BEZ235 (500 nM) as described in the Materials and Methods. Flow cytometric analysis showed that 24-hour treatment with rapamycin and everolimus significantly induced apoptosis in PCK cholangiocytes, while it was significantly inhibited by the treatment with NVP-BEZ235 (A). NVP-BEZ235 significantly reduced the expression of cleaved caspase 3 in PCK cholangiocytes that was determined using Western blot analysis (B). The results of the semiquantitative analysis of Western blotting are shown in C. Flow cytometric analysis showed that silencing of caspase 3 using siRNA in PCK cholangiocytes resulted in the reduction of the percentage of apoptotic cells under normal and H_2_O_2_-treated conditions (D). The analysis with quantitative real-time PCR showed that treatment with NVP-BEZ235 significantly increased the expression of bcl-2 mRNA in PCK cholangiocytes (E). *, *p*<0.01; **, *p*<0.05 (vs. untreated control).

Apoptosis was determined following the treatment of PCK cholangiocytes with rapamycin, everolimus and NVP-BEZ235. Flow cytometric analysis showed that rapamycin and everolimus significantly induced apoptosis in PCK cholangiocytes (Figure 3A). By contrast, apoptosis was significantly inhibited by the treatment with NVP-BEZ235 (Figure 3A).

In relation to the inhibition of apoptosis, NVP-BEZ235 significantly reduced the expression of cleaved caspase 3 in PCK cholangiocytes, while it tended to be unchanged or rather increased following the treatment with everolimus (Figure 3B and 3C). Silencing of caspase 3 using siRNA in PCK cholangiocytes reduced the percentage of apoptotic cells from 10.04% to 7.72% under normal condition, and from 45.16% to 25.28% under H_2_O_2_-treated condition, confirming that caspase 3 was involved in the induction of apoptosis in PCK cholangiocytes (Figure 3D). In consistent with the inhibition of apoptosis by NVP-BEZ235, the analysis with quantitative real-time PCR showed that treatment with NVP-BEZ235 significantly increased the expression of bcl-2 mRNA in PCK cholangiocytes (Figure 3E).

### Effects of mTOR Inhibitors on Cholangiocyte Autophagy in vitro

**Figure 4:**
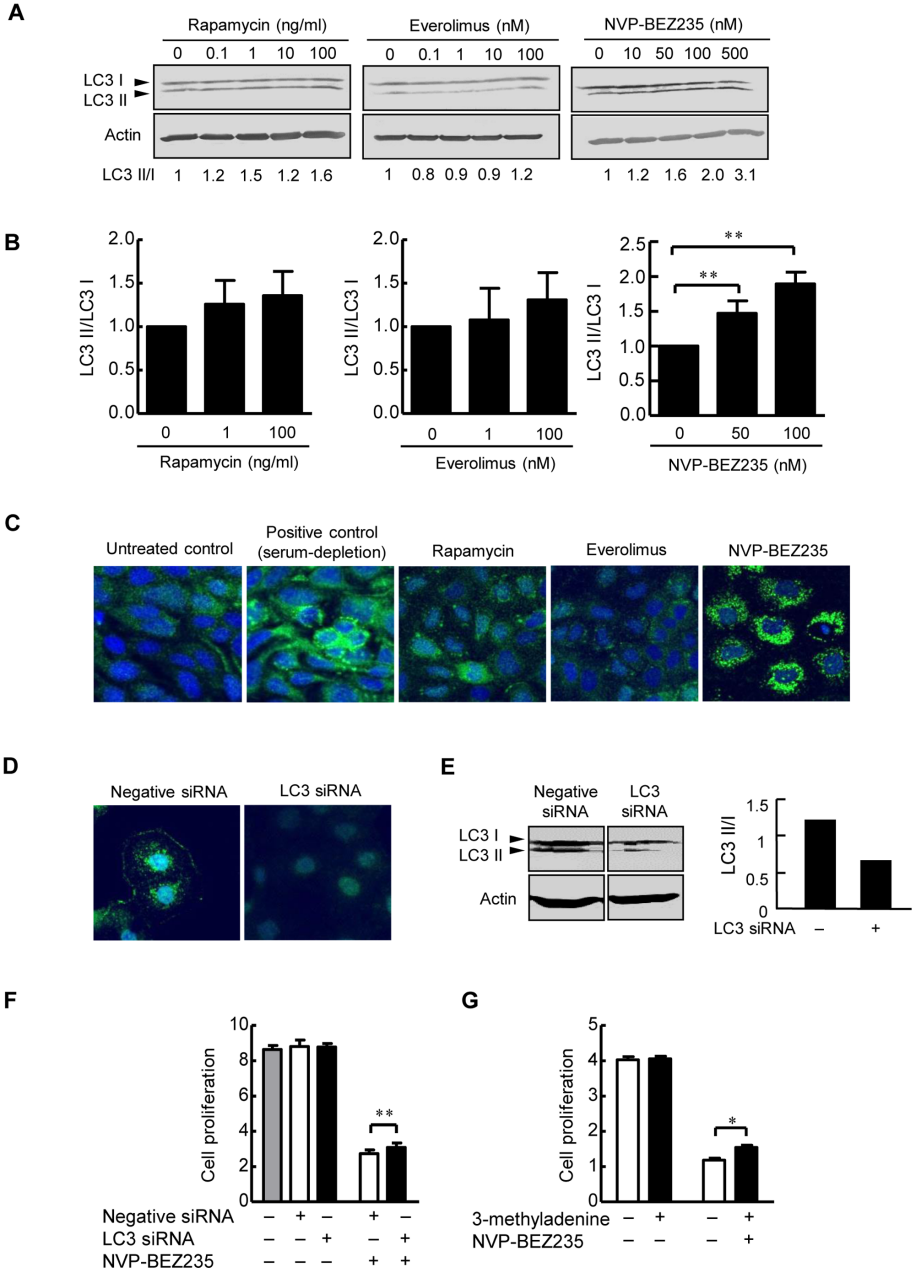
Effects of mTOR inhibitors on cholangiocyte autophagy in vitro. PCK cholangiocytes were treated with rapamycin, everolimus and NVP-BEZ235 for 24 hours, and autophagy of the cells was detected by the conversion of LC3 I to LC3 II using Western blot analysis. NVP-BEZ235 induced a dose-dependent increase in the autophagy-specific LC3 II form, while rapamycin and everolimus did not significantly induce autophagy in PCK cholangiocytes (A). The results of the semiquantitative analysis of Western blotting are shown in B. Immunocytochemistry of LC3 showed the induction of autophagy by NVP-BEZ235 in PCK cholangiocytes (C). Silencing of LC3 was performed using LC3 siRNA in PCK cholangiocytes, and immunocytochemistry (D) and Western blot analysis (E) showed that LC3 siRNA reduced LC3 II formation in the cholangiocytes. When PCK cholangiocytes were treated with NVP-BEZ235 (100 nM) for 48 hours, the cell proliferative activity of the LC3 siRNA-treated cells was significantly high than those without LC3 siRNA treatment that was determined using the WST-1 assay (F). Following 48-hour treatment of PCK cholangiocytes with 3-methyladenine (500 nM) together with NVP-BEZ235 (100 nM), the cell proliferative activity was significantly increased compared to those with NVP-BEZ235 alone (G). Original magnifications, x1000 (C and D). *, *p*<0.01; **, *p*<0.05 (vs. untreated control).

The occurrence of autophagy in cholangiocytes was detected by the conversion of LC3 I to LC3 II. Western blot analysis showed that NVP-BEZ235 induced a dose-dependent increase in the autophagy-specific LC3 II form, while rapamycin and everolimus did not significantly induce autophagy in PCK cholangiocytes (Figure 4A and 4B). Immunocytochemistry of LC3 confirmed the induction of autophagy by NVP-BEZ235 (Figure 4C).

To further determine the involvement of autophagy in the inhibition of the cell proliferative activity by NVP-BEZ235, silencing of LC3 was performed using LC3 siRNA in PCK cholangiocytes, and the cell proliferative activity was compared for the cells with and without NVP-BEZ235 treatment. The gene silencing was monitored using immunocytochemistry and Western blotting for PCK cholangiocytes maintained with serum-free medium, and the analysis showed that LC3 siRNA reduced LC3 II formation in the cholangiocytes, although their expression was not completely diminished (Figure 4D and 4E). As expected, when PCK cholangiocytes were treated with NVP-BEZ235, the cell proliferative activity of the LC3 siRNA-treated cells was significantly high than those without LC3 siRNA treatment (Figure 4F). When PCK cholangiocytes were treated with 3MA, a specific inhibitor of autophagy, together with NVP-BEZ235, the cell proliferative activity was significantly increased compared to those with NVP-BEZ235 alone (Figure 4G), confirming that autophagy was involved in the growth inhibition by NVP-BEZ235 in PCK cholangiocytes.

### Inhibition of Biliary Cyst Formation by mTOR Inhibitors in vitro

**Figure 5:**
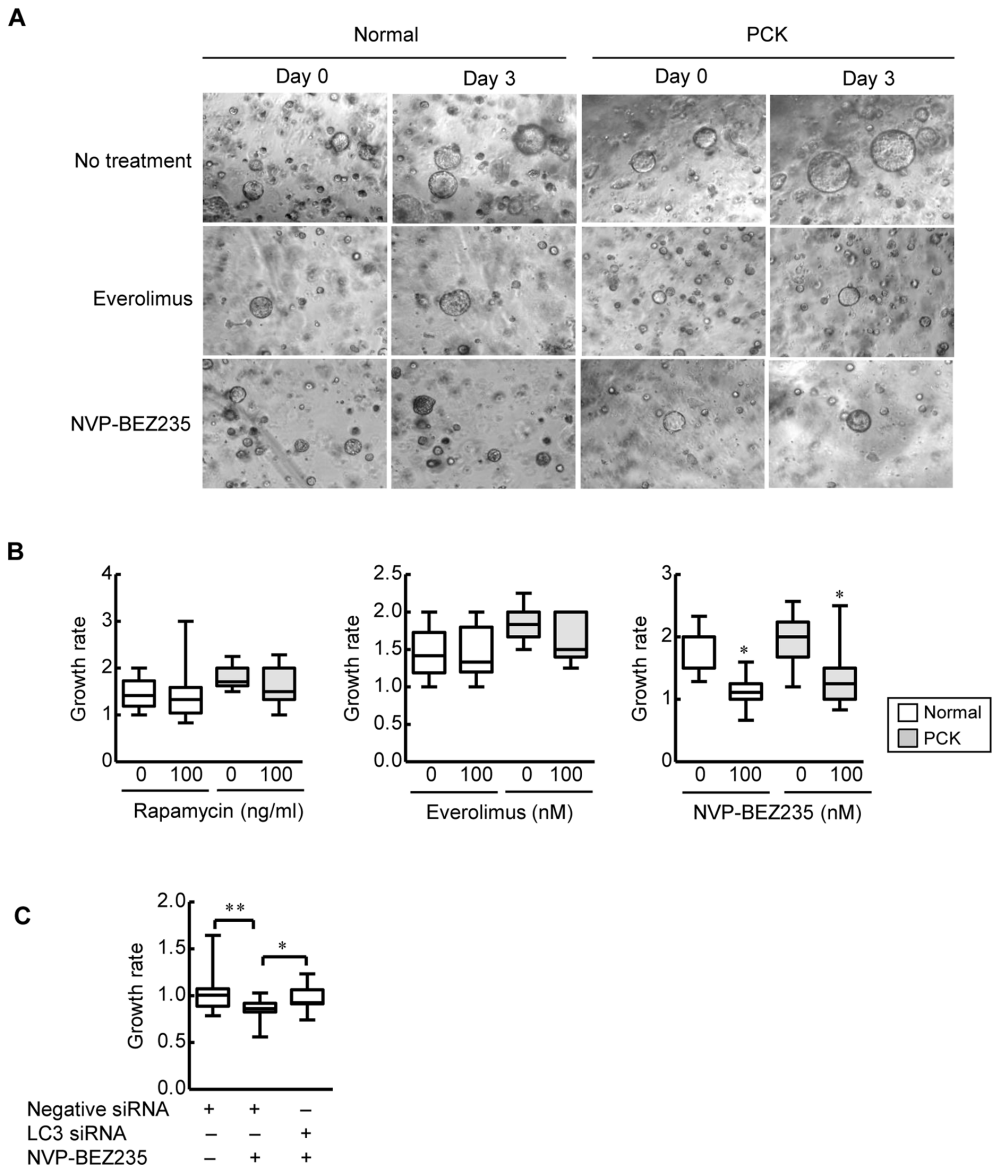
Inhibition of biliary cyst formation by mTOR inhibitors in vitro. The effects of mTOR inhibitors on biliary cyst formation were examined for normal and PCK cholangiocytes using three-dimensional cell culture system as described in the Materials and Methods. PCK cholangiocytes grew more rapidly in a spheroidal form in the Matrigel compared to normal cholangiocytes under normal condition (A). NVP-BEZ235 significantly inhibited cystic growth of both normal and PCK cholangiocytes, while rapamycin and everolimus did not have significant inhibitory effects on biliary cyst formation of the cholangiocytes (B). Knockdown of LC3 with siRNA of PCK cholangiocytes that were treated with NVP-BEZ235 significantly increased the biliary cyst formation (C). *, *p*<0.01 (vs. untreated control) (B): *, *p*<0.01; **, *p*<0.05 (C).

The effects of mTOR inhibitors on biliary cyst formation were examined using three-dimensional cell culture system. PCK cholangiocytes grew more rapidly in a spheroidal form in the Matrigel compared to normal cholangiocytes under normal condition (Figure 5A). NVP-BEZ235 significantly inhibited cystic growth of both normal and PCK cholangiocytes (Figure 5A and 5B). Knockdown of LC3 with siRNA of PCK cholangiocytes that were treated with NVP-BEZ235 significantly increased the biliary cyst formation (Figure 5C), showing further evidence of the involvement of autophagy in these processes. By contrast, rapamycin and everolimus did not have significant inhibitory effects on biliary cyst formation of both normal and PCK cholangiocytes (Figure 5A and 5B).

### In vivo Administration of NVP-BEZ235

**Figure 6:**
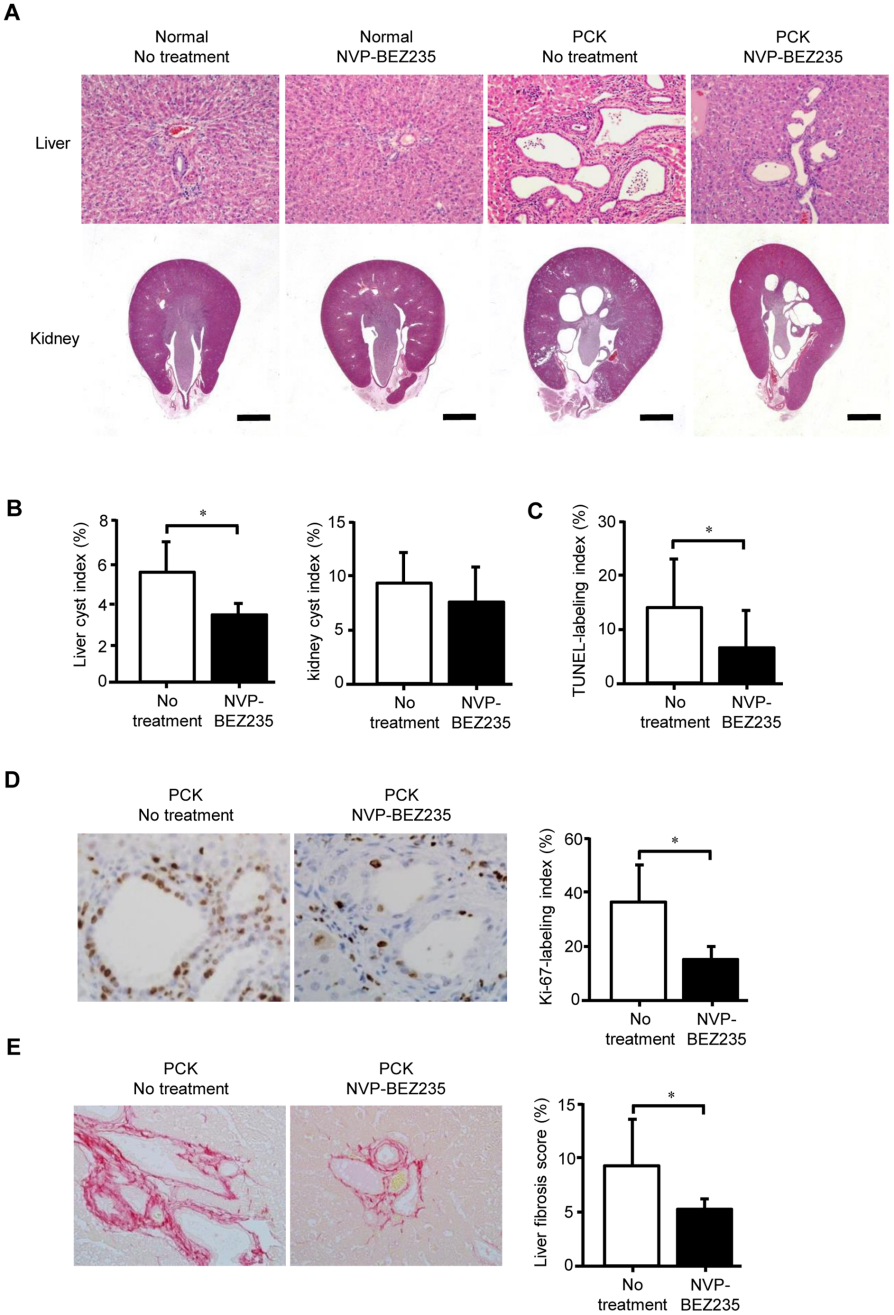
Effects of in-BEZ235 on liver and renal diseases of the PCK rat. Normal and PCK rats were treated with NVP-BEZ235 or vehicle alone daily between 4 and 8 weeks of age. Liver and kidney sections stained with hematoxylin-eosin showed that NVP-BEZ235 improved dilatation of intrahepatic bile ducts of the PCK rat, but no beneficial effects were observed on kidney lesions (A). For the PCK rat, liver and kidney cyst index (B), TUNEL-labeling index of the biliary epithelial cells (C), Ki-67-labeling index of the biliary epithelial cells (D), and liver fibrosis score (E) were determined as described in the Materials and Methods. Treatment with NVP-BEZ235 significantly reduced liver cyst index, TUNEL-labeling index, Ki-67-labeling index and liver fibrosis score of the PCK rat, whereas kidney cyst index was unaffected. Original magnifications: x200 (A, upper panel; E); x400 (D). Bars, 2 mm (A, lower panel). *, *p*<0.01.

Finally, the effects of in vivo administration of NVP-BEZ235 on the progression of liver and renal diseases in the PCK rat were examined. Hematological analysis showed that liver and renal functions were preserved following the treatment, but retardation in body weight increase was noted in both normal and PCK rats by the treatment (Table 1). As expected, the treatment reduced the extent of dilatation of intrahepatic bile ducts of the PCK rat, whereas no apparent histological changes were observed in the liver of normal rats (Figure 6A). On the contrary, no beneficial effects were observed on renal cyst development because of the treatment as evaluated using routine histological sections (Figure 6A). Histomorphometric analysis using whole liver and kidney tissues confirmed that the treatment significantly reduced liver cyst index, but not kidney cyst index (Figure 6B).

**Table 1:** Treatment of normal and PCK rats with NVP-BEZ235.

	Normal no treatment	Normal NVP-BEZ235	PCK no treatment	PCK NVP-BEZ235
Number of rats	6	5	5	6
Body weight (g)	340±5	275±7*	337±11	291±5**
Liver/body weight (%)	4.4±0.1	4.3±0.3	6.1±0.5	5.6±0.1
Kidney/body weight (%)	0.86±0.02	0.84±0.03	1.19±0.10	1.03±0.00
Aspertate aminotransferase (IU/L)	85±3	94±5	91±14	88±6
Alkaline phosphatase (U/L)	1413±111	1040±169	1174±114	994±48
Total protein (g/dL)	5.9±0.1	5.8±0.0**	5.5±0.3	5.7±0.1
Albumin (g/dL)	4.5±0.1	4.4±0.1	3.2±0.1	3.9±1.0*
Blood urea nitrogen (mg/dL)	17±1	16±2	20±2	17±0
Cretinine (mg/dL)	0.30±0.00	0.32±0.02	0.40±0.03	0.32±0.02

* , *p*<0.01;

** , *p*<0.05 (vs. untreated control).

In the liver of the PCK rat, apoptosis of biliary epithelial cells was significantly inhibited by the treatment (Figure 6C), while the cell proliferative activity of the cells was significantly reduced (Figure 6D), which were in consistent with the results of in vitro experiments. In addition, the treatment significantly improved the extent of liver fibrosis of the PCK rat (Figure 6E).

**Figure 7:**
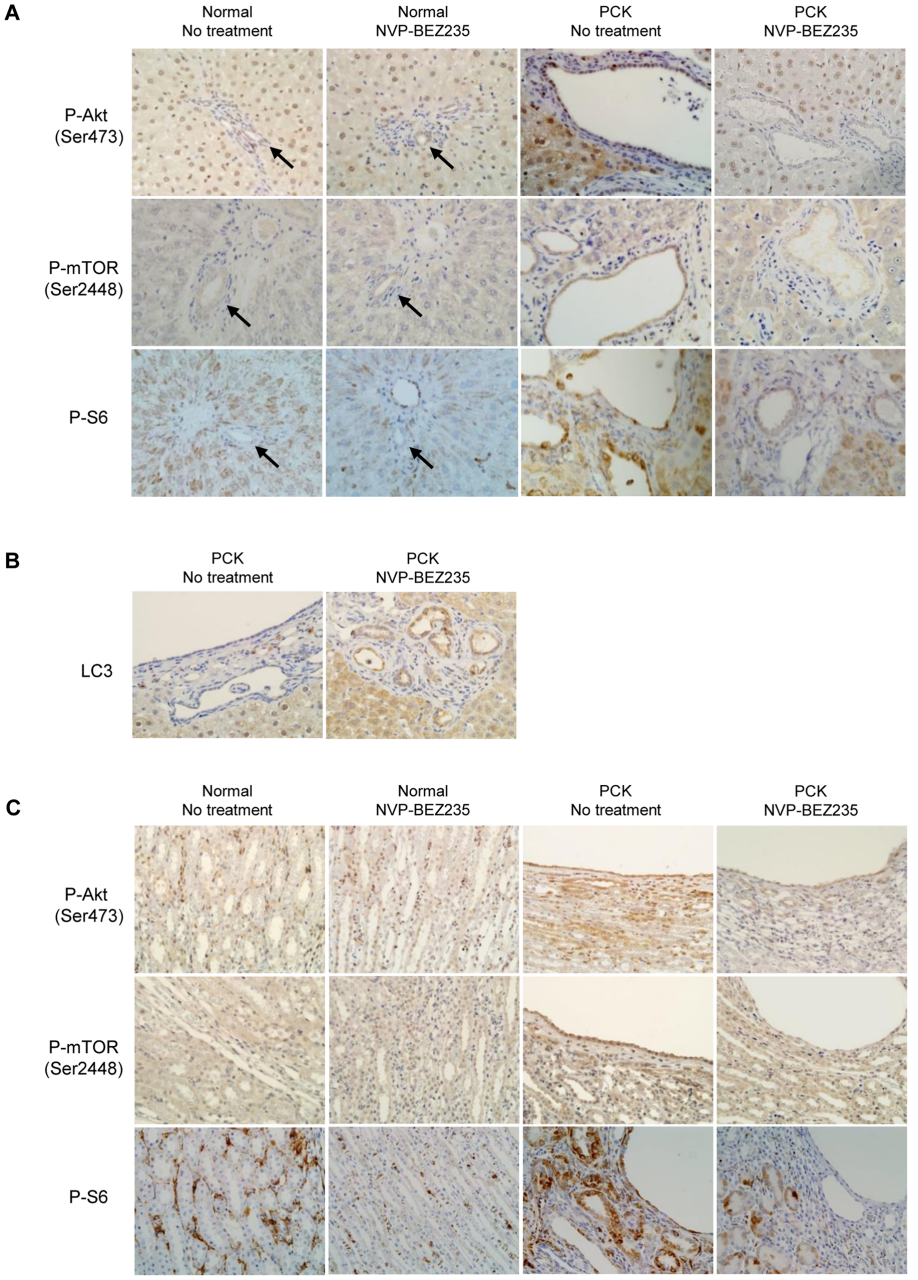
Immunohistochemical analysis of the the liver and kidney of the PCK rat treated with NVP-BEZ235. Treatment with NVP-BEZ235 reduced the expression of p-Akt (Ser473), p-mTOR (Ser2448) and p-S6 in bile duct epithelium of the PCK rat (A). The treatment increased immunohistochemical expression of LC3 in bile duct epithelium of the PCK rat (B). In the kidney, the expression of p-Akt (Ser473), p-mTOR (Ser2448) and p-S6 was increased in collecting tubule-derived cyst epithelium of the PCK rat without NVP-BEZ235 treatment compared to those in the renal tubules of normal rat, and their expression appeared to be reduced in the kidney of the PCK rat following the treatment (C). Arrows indicate interlobular bile ducts of the normal liver. Original magnifications; x400 (A–C).

Immunohistochemical analysis confirmed that the expression of p-Akt (Ser473), p-mTOR (Ser2448) and p-S6 was reduced in bile duct epithelium of the PCK rat treated with NVP-BEZ235 (Figure 7A). The treatment increased immunohistochemical expression of LC3 in bile duct epithelium of the PCK rat (Figure 7B), suggesting the occurrence of autophagy.

In the kidney, the expression of p-Akt (Ser473), p-mTOR (Ser2448) and p-S6 was increased in collecting tubule-derived cyst epithelium of the PCK rat without NVP-BEZ235 treatment compared to those in the renal tubules of normal rat (Figure 7C). Although the treatment had no significant effect on kidney cyst index, the immunohistochemical expression of these molecules appeared to be reduced in the kidney of the PCK rat following the treatment (Figure 7C).

## Discussion

This study demonstrated that the PI3K/Akt/mTOR pathway was activated in PCK cholangiocytes. The treatment with NVP-BEZ235 effectively inhibited cholangiocyte cystic proliferation by enhancing autophagy and inhibiting apoptosis in vitro, and the inhibitory effects were also observed in the PCK rat treated with NVP-BEZ235 in vivo. Rapamycin and everolimus induced apoptosis of the cells, but they could not inhibit cystic growth of the cells in the three-dimensional cell culture system.

A recent study showed that in vivo administration of rapamycin failed to inhibit cystic dilatation of the bile ducts of the PCK rat, and the authors considered that this might be due to intrinsic or acquitted rapamycin resistance [14]. In clinical trials where rapamycin and everolimus did not show beneficial effects in patients with ADPKD, low tissue concentrations of the drugs at clinically tolerable doses have been considered as one of the causes [12], [13]. Our data suggest that the inhibition of mTORC1 alone may be insufficient for the inhibition of cell proliferation of PCK cholangiocytes, because other complementary signaling pathways were activated in PCK cholangiocytes. More prominent inhibitory effects of NVP-BEZ235 rather than rapamycin and everolimus on the cell proliferation of PCK cholangiocytes support that the inhibition of other signaling pathways such as PI3K may be important for inhibiting the cell proliferative activity.

Drug-induced growth inhibition is often associated with increased apoptosis in PKD cells, and apoptosis has been implicated in strategies for therapeutic intervention [21]. However, the studies of the effects of rapamycin on apoptosis in PKD are conflicting [26], and the present study showed that rapamycin and everolimus induced apoptosis in PCK cholangiocytes. By contrast, NVP-BEZ235 inhibited apoptosis by reducing the expression of cleaved caspase 3 and by inducing bcl-2 expression. In several cancer cell lines, it has been shown that NVP-BEZ235 induced apoptosis and cell cycle arrest accompanied by increased caspase 3 activity, and bcl-2 abrogated the effects of NVP-BEZ235 [27]–[29]. Regarding the effects of NVP-BEZ235 on apoptosis in PKD cells, limited data are available, and further detailed studies are required to clarify the mechanism of the inhibition of apoptosis in PCK cholangiocytes by NVP-BEZ235.

In the kidney, a recent study showed an increase in LC3 II and beclin 1 expression in the late stages of PKD in rodent models of ADPKD [22]. In consistent with the facts that autophagy has been described as hypoxia-inducible factor-1α-dependent adaptive response, HIF-1α was also highly expressed in the late stages of PKD in relation to the occurrence of autophagy [22]. In their studies, however, the hypoxia-inducible factor-1α inhibitor had no significant effects on kidney lesions in vivo. To date, published data on the involvement of autophagy is unavailable for the disease pathogenesis of kidney and liver lesions of ARPKD as well as Caroli’s disease and their rodent models.

The mTOC1 is a negative regulator of autophagy, directly by phosphorylating ULK1 and preventing ULK1-Atg13-FIP200 complex formation, and indirectly by phosphorylating S6 and 4E-BP1 [30]. Recently, reports on the involvement of mTORC2 in autophagy have started to emerge, and mTORC2 regulates autophagy by affecting forkhead box O and regulating some key autophagy genes such as LC3 and beclin 1 [31]. In these conditions, rapamycin is unable to induce autophagy. The regulation of autophagy process by mTORC1/2 suggests that the inhibition of mTOR kinase activity, by having an impact on both mTOR complexes, may have a greater effect on autophagy, compared to rapamycin alone. Indeed, NVP-BEZ235, not rapamycin and everolimus, significantly induced autophagy in PCK cholangiocytes.

In this study, rapamycin, everolimus and NVP-BEZ235 blocked the phosphorylation of Akt (Ser473) and the downstream effectors of mTORC1/2, S6 and 4E-BP1, in PCK cholangiocytes. The inhibition of the phosphorylation of Akt (Ser473) by the mTORC1 inhibitors was consistent with the reported findings showing that higher concentration of rapamycin might target mTORC2 [32]. It was of note that higher concentration (500 nM) of NVP-BEZ235 markedly reduced the expression of p-Akt (Ser473) and p-4E-BP1 in PCK cholangiocytes. Since recent studies have shown that 4E-BP1 is a potential new target molecule for anti-cancer therapy [33], the marked inhibitory effects of NVP-BEZ235 on the phosphorylation of 4E-BP1 as well as Akt (Ser473) further support its applicability as a therapeutic agent.

As expected, the treatment of the PCK rats with NVP-BEZ235 significantly inhibited cystic dilatation of the intrahepatic bile ducts in vivo, whereas renal cyst development was not affected by the treatment. Similarly, an epidermal growth factor tyrosine kinase inhibitor (gefitinib) significantly has been shown to reduce cystic bile duct dilatation of the PCK rat in vivo, but kidney lesions were unaffected or rather worsened according to our previous study [34]. These results suggest that the mechanism of cyst progression of the PCK rat may be different between the liver and kidney. In addition, in vivo administration of NVP-BEZ235 caused retardation in body weight increase in the rats, indicating the limitation of clinical application of this agent for the patients with ARPKD, at least at the dosage and the treatment period used in this study.

In conclusion, this study showed the involvement of aberrant activation of the PI3K/mTOR pathway in the dilatation of intrahepatic bile ducts of the PCK rat. Although the PI3K/Akt/mTOR signaling pathway represents a complex network, inhibition of the pathway using specific inhibitors attenuates cystic proliferation of PCK cholangiocytes via the mechanism involving apoptosis and/or autophagy of the cells, which is preferably further addressed for therapeutic purpose.
